# Rapidly increasing cumulative incidence of coronavirus disease (COVID-19) in the European Union/European Economic Area and the United Kingdom, 1 January to 15 March 2020

**DOI:** 10.2807/1560-7917.ES.2020.25.11.2000285

**Published:** 2020-03-19

**Authors:** Pete Kinross, Carl Suetens, Joana Gomes Dias, Leonidas Alexakis, Ariana Wijermans, Edoardo Colzani, Dominique L. Monnet, European Centre for Disease Prevention and Control (ECDC) Public Health Emergency Team

**Affiliations:** 1 European Centre for Disease Prevention and Control, Solna, Sweden; 2 The members of the ECDC Public Health Emergency Team are listed at the end of this article

**Keywords:** SARS-CoV-2, COVID-19, coronavirus disease, coronavirus, EU/EEA, United Kingdom, Europe

## Abstract

The cumulative incidence of coronavirus disease (COVID-19) cases is showing similar trends in European Union/European Economic Area countries and the United Kingdom confirming that, while at a different stage depending on the country, the COVID-19 pandemic is progressing rapidly in all countries. Based on the experience from Italy, countries, hospitals and intensive care units should increase their preparedness for a surge of patients with COVID-19 who will require healthcare, and in particular intensive care.

On 31 December 2019, a cluster of pneumonia cases of unknown aetiology was reported in Wuhan, Hubei Province, China. On 9 January 2020, the China Center for Disease Control and Prevention reported the causative agent as being a novel coronavirus now referred to as severe acute respiratory syndrome coronavirus 2 (SARS-CoV-2) [1]. Since, the illness resulting from SARS-CoV-2 infection has been named coronavirus disease (COVID-19). Evidence to date is that ca 80% of individuals with COVID-19 have a mild disease, i.e. a respiratory tract infection with or without pneumonia, and most of these recover [1]. In ca 14% cases, COVID-19 develops into a more severe disease requiring hospitalisation while the remaining 6% cases experience critical illness requiring intensive care. The mortality of patients hospitalised due to COVID-19 is ca 4% [1]. In this study, we assess the trends in the cumulative incidence of COVID-19 in each European Union/European Economic Area (EU/EEA) country and the United Kingdom (UK) and compare them to that of Hubei Province, China. We also compare the current number of COVID-19 cases in EU/EEA countries and the UK with that in Italy during 31 January–15 March 2020.

## COVID-19 cases in EU/EEA countries and the UK

Subsequent to China, COVID-19 underwent further geographical spread and the dynamic of the COVID-19 pandemic in the rest of the world currently follows that of this country [2]. On 11 March 2020, the Director General of the World Health Organization (WHO) declared COVID-19 a pandemic [3]. In the 5 March issue of *Eurosurveillance *2020, Spiteri et al. reported on the first European confirmed COVID-19 cases according to the WHO case definition [4,5]. In the EU/EEA, the first three confirmed cases were reported by France on 24 January 2020 in persons returning from Wuhan, Hubei Province, China [2]. As at 15 March 2020, COVID-19 cases had been detected in all 30 EU/EEA countries and the United Kingdom (UK) [6], whereby between 31 December 2019 and that date included, 39,768 cases and 1,727 deaths had been reported, with 17,750 cases and 1,441 deaths from Italy alone [6].

## Obtaining cumulative number and cumulative incidence of COVID-19 cases 

At the European Centre for Disease Prevention and Control (ECDC), the notified COVID-19 case counts in each country worldwide, obtained from only official sources such as the countries’ Ministry of Health, national and regional health authorities and the WHO, are updated each day at 8:00 a.m. These data were used for assessing the trends of COVID-19 in EU/EEA and the UK, and comparing them to that in Italy. As a proxy of the prevalence of active COVID-19 cases, we calculated the 14-day truncated cumulative incidence of COVID-19 cases, thus taking into account the natural course of COVID-19, in each EU/EEA country and the UK, during the 1 January–15 March 2020 period. We also presented the cumulative number of notified cases of each country as at 15 March 2020 8:00 a.m. compared with that of Italy for the 31 January–15 March 2020 period.

## Trends of COVID-19 in EU/EEA countries and the UK

**Figure 1:**
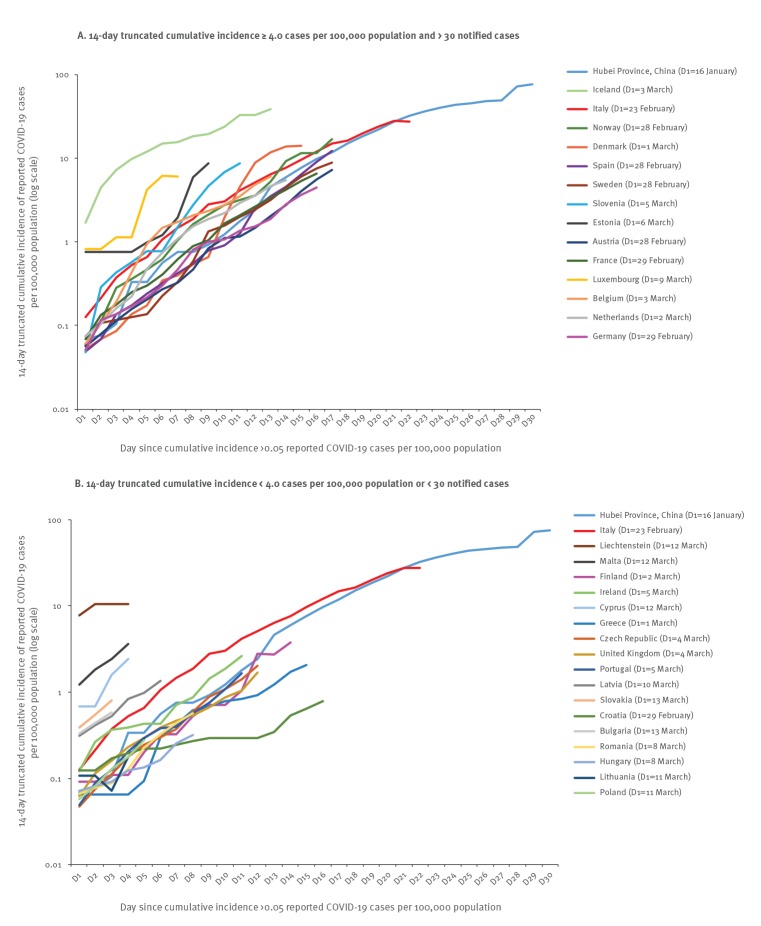
Time distribution of the 14-day truncated cumulative incidence of COVID-19 for (A) 14-day truncated cumulative incidence ≥ 4.0 cases per 100,000 population and > 30 notified cases^a^ and (B) 14-day truncated cumulative incidence < 4.0 cases per 100,000 population or < 30 notified cases^b^, EU/EEA countriesand the UK^c^, 15 March 2020

COVID-19: coronavirus disease; EU/EEA: European Union/European Economic Area; UK: United Kingdom.

^a ^The 14-day truncated cumulative incidence of COVID-19 cases distribution in each country is compared with that of Hubei Province, China.

^b ^The 14-day truncated cumulative incidence of COVID-19 cases distribution in each country is compared with that of Italy (which, unlike the other countries in this panel, has a 14-day truncated cumulative incidence of ≥ 4.0 cases per 100,000 population and > 30 notified cases as at 15 March) and of Hubei Province, China.

^c^ The 14-day truncated cumulative incidence of COVID-19 cases is shown from the day (D1) a country reported a 14-day truncated cumulative incidence > 0.05 cases per 100,000 population.

**Figure 2:**
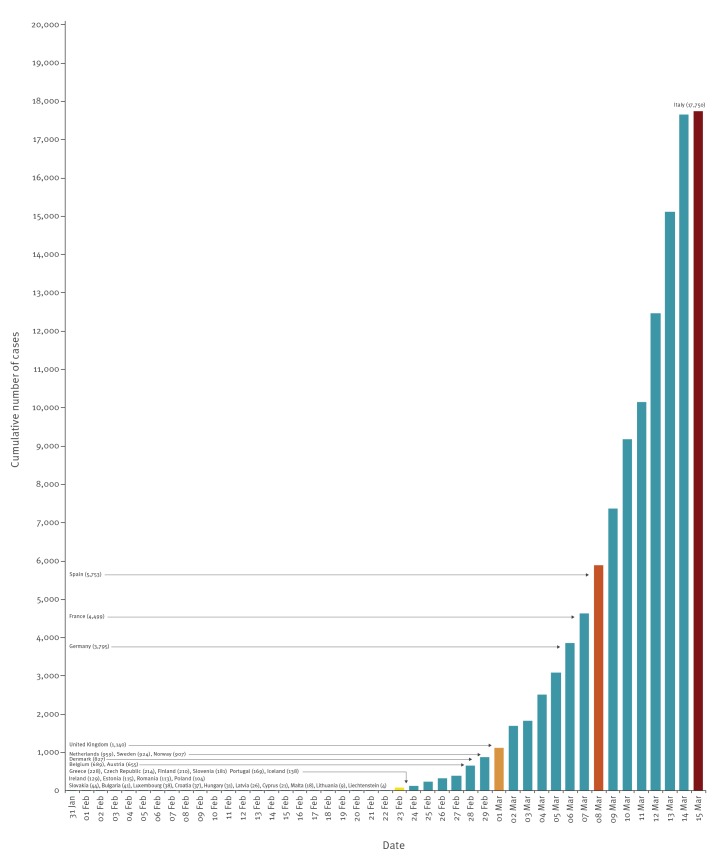
Cumulative number of COVID-19 cases in EU/EEA countries and the UK as at 15 March compared with that in Italy, 31 January–15 March 2020 (total number of cases in the EU/EEA and the UK as at 15 March 2020 8:00 a.m. = 39,768)

COVID-19: coronavirus disease; EU/EEA: European Union/European Economic Area; UK: United Kingdom.

The trends in the 14-day truncated cumulative incidence of COVID-19 cases in EU/EEA countries and the UK generally followed that of Hubei Province (China) (Figure 1). For the EU/EEA and the UK overall, the cumulative incidence of COVID-19 started to increase around 21 February and then increased sharply around 28 February 2020 (Supplementary material). This was mostly driven by the rapid increase in the number of reported cases from Italy, but all other EU/EEA countries and the UK showed similar increasing trends of the cumulative incidence of COVID-19 (Supplementary material). Figure 2 shows the cumulative number of COVID-19 cases, in EU/EEA countries and the UK compared with that in Italy for the 31 January–15 March 2020 period. It highlights that, as at 15 March 8:00 a.m., 15 other EU/EEA countries and the UK had already reported a total number of cases comparable to that of Italy just 3 weeks prior or less.

The crimson red bar indicates the position of Italy as at 15 March, while the red, orange and yellow bars indicate the position of Italy 1, 2 and 3 weeks prior, respectively. The number of cases in each country as at 15 March is indicated in parentheses.

## Discussion

Our results indicate that the number of notified cases of COVID-19 is rapidly increasing in the EU/EEA and the UK. The observed trends in the cumulative incidence of COVID-19 suggest that the pandemic is progressing at a comparable speed in all countries. This is despite countries being at different stages, variations in national public health responses, and possibly different case definitions in countries and different protocols for selecting patients that must be tested for confirmation of COVID-19, including catch-up testing.

Early March 2020, doctors in the affected regions of Italy described a situation in which ca 10% of patients with COVID-19 required intensive care [7] and media sources reported that hospitals and intensive care units in these regions had already reached their maximum capacity [8=^13^]. Data on admission of COVID-19 cases in a hospital and/or an intensive care unit are currently available at EU/EEA level for only 6% and 1% cases, respectively (data not shown). They should, however, be collected in a systematic fashion to complement current surveillance data that focus on the number of reported cases and the number of deaths. A study performed in 2010–11 showed a large variation in the availability of intensive care and intermediate care beds in Europe, ranging from 29.2 in Germany to 4.2 beds per 100,000 population in Portugal [14]. This means that countries may have more or less resources than Italy (12.5 intensive care and intermediate care beds per 100,000 population in 2010–11). Modelling scenarios related to healthcare capacity saturation, with estimates for each EU/EEA country and the UK of the prevalence of hospitalised COVID-19 cases associated with a > 90% risk of exceeding intensive care bed capacity, are provided in the sixth update of the ECDC rapid risk assessment on COVID-19 [1]. Since cases have so far clustered in certain regions in EU/EEA countries and the UK, and hospitals and intensive care units usually serve a defined regional catchment population, information about cases and intensive care beds should preferably be made available at the Nomenclature of territorial units for statistics 2 (NUTS-2) level.

The experience from Italy and the current trends in other countries show that the COVID-19 pandemic is progressing rapidly in the EU/EEA and the UK. Countries, hospitals and intensive care units should thus prepare themselves for a scenario of sustained community transmission of SARS-CoV-2 and an increase in the number of patients with COVID-19 requiring healthcare, and in particular intensive care, such as the one occurring in the affected regions of Italy. As pointed out in the recent ECDC rapid risk assessment, a rapid, proactive and comprehensive approach is essential to delay the spread of SARS-COV-2, with a shift from a containment to a mitigation approach, as the anticipated rapid increase in the number of cases may not provide decision makers and hospitals enough time to comprehend, accept and adapt their response accordingly if not implemented ahead of time [1]. The rapid risk assessment also lists the public health measures to mitigate the impact of the pandemic. There is a short window of opportunity during which countries have the possibility to further increase their control efforts to slow down the spread of SARS-CoV-2 and decrease the pressure on healthcare. Failing this, it is likely that the healthcare systems of other EU/EEA countries will face a surge of patients that require intensive care within the coming days or weeks.

## Editorial note

This article was published as an e-Alert on 16 March 2020.

## ECDC Public Health Emergency Team

Cornelia Adlhoch, Agoritsa Baka, Julien Beauté, Jordi Borrell Pique, Eeva Broberg, Sergio Brusin, Nick Bundle, Mike Catchpole, Orlando Cenciarelli, Scott Chiossi, Bruno Ciancio, Catia Cunha, Angelo D'Ambrosio, Stefania De Angelis, Dragoslav Domanović, Margot Einöder-Moreno, Laura Espinosa, Emilie Finch, Silvia Funke, Céline Gossner, Gaëtan Guyodo, Joana Haussig, Hector Honrubia, Josep Jansa, Helen Johnson, Tommi Kärki, John Kinsman, Csaba Ködmön, Favelle Lamb, Katrin Leitmeyer, Felix Lötsch, Otilia Mårdh, Angeliki Melidou, Hanna Merk, Grazina Mirinaviciutė, Thomas Mollet, Lina Nerlander, Teymur Noori, Daniel Palm, Pasi Penttinen, Anastasia Pharris, Diamantis Plachouras, Emmanuel Robesyn, Senia Rosales-Klintz, Andreea Salajan, Gianfranco Spiteri, Svetla Tsolova.

## Supplementary Data

422000 Supplementary Material

## References

[1] European Centre for Disease Prevention and Control (ECDC). Novel coronavirus disease 2019 (COVID-19) pandemic: increased transmission in the EU/EEA and the UK – sixth update, 12 March 2020. ECDC: Stockholm; 2020. Available from: https://www.ecdc.europa.eu/sites/default/files/documents/RRA-sixth-update-Outbreak-of-novel-coronavirus-disease-2019-COVID-19.pdf

[2] Bernard Stoecklin S Rolland P Silue Y Mailles A Campese C Simondon A Investigation Team First cases of coronavirus disease 2019 (COVID-19) in France: surveillance, investigations and control measures, January 2020. Euro Surveill. 2020;25(6):2000094. 10.2807/1560-7917.ES.2020.25.6.2000094 32070465 PMC7029452

[3] WHO Director-General's opening remarks at the media briefing on COVID-19 - 11 March 2020. World Health Organization: Geneva; 2020. Available from: https://www.who.int/dg/speeches/detail/who-director-general-s-opening-remarks-at-the-media-briefing-on-covid-19---11-march-2020

[4] Spiteri G Fielding J Diercke M Campese C Enouf V Gaymard A First cases of coronavirus disease 2019 (COVID-19) in the WHO European Region, 24 January to 21 February 2020. Euro Surveill. 2020;25(9):2000178. 10.2807/1560-7917.ES.2020.25.9.2000178 32156327 PMC7068164

[5] World Health Organization (WHO). Coronavirus disease (COVID-19) technical guidance: Surveillance and case definitions. Geneva: WHO; 2020. Available from: https://www.who.int/emergencies/diseases/novel-coronavirus-2019/technical-guidance/surveillance-and-case-definitions

[6] European Centre for Disease Prevention and Control. Situation update as of 15 March 2020 08:00. Distribution of COVID-19 cases worldwide, as of 15 March 2020. [Accessed 15 Mar 2020]. Available from: https://www.ecdc.europa.eu/en/geographical-distribution-2019-ncov-cases

[7] European Society of Intensive Care Medicine. Shared experience & guidance from our colleagues in Northern Italy. 5 March 2020. [Accessed 9 March 2020]. Available from: https://www.esicm.org/covid-19-update-from-our-colleagues-in-northern-italy/

[8] Baldi C. Allarme dei sanitari Lombardi: “Le nostre strutture sottoposte aa pressione superior a ogni possibilità di riposte” [Alarm from healthcare professionals in Lombardy: “Our structures are under a pressure which is superior to any possibility of response”]. La Stampa, 8 March 2020. Italian. [Accessed 9 Mar 2020]. Available from: https://www.lastampa.it/topnews/primo-piano/2020/03/08/news/allarme-dei-sanitari-lombardi-le-nostre-strutture-sottoposte-a-pressione-superiore-a-ogni-possibilita-di-risposta-1.38566390

[9] Bocci M. Coronavirus, l'anestesita Petrini: “Oggi la scelta di chi curare richiede regole certe” [Coronavirus, the anaestesiologist Petrini: “Today the choice of whom to cure requires clear recommendations“]. La Repubblica, 8 March 2020. Italian. [Accessed 9 March 2020]. Available from: https://www.repubblica.it/cronaca/2020/03/08/news/petrini_oggi_la_scelta_di_chi_curare_richiede_regole_certe_-250594687/

[10] Chi. Bal. Una tenda davanti all’ingresso. Cremona, ospedale al collasso: “Non c’è spazio per i pazienti” [A tent in front of the entrance. Cremona, hospital facing collapse: “We do not have room for the patients”]. La Stampa, 8 March 2020. Italian. [Accessed 9 Mar 2020]. Available from: https://www.lastampa.it/cronaca/2020/02/28/news/una-tenda-davanti-all-ingresso-cremona-ospedale-al-collasso-non-c-e-spazio-per-i-pazienti-1.38525490

[11] Imarisio M. Coronavirus, il medico di Bergamo: «Negli ospedali siamocome in guerra. A tutti dico: state a casa» [Coronavirus, a doctor from Bergamo: «In the hospitals we are like in a war scene. I am telling everyone: stay home»]. Corriere della Serra, 8 March 2020. Italian. [Accessed 9 Mar 2020]. Available from: https://www.corriere.it/cronache/20_marzo_09/coronavirus-scegliamo-chi-curare-chi-no-come-ogni-guerra-196f7d34-617d-11ea-8f33-90c941af0f23_preview.shtml?reason=unauthenticated&cat=1&cid=sTfRVocj&pids=FR&credits=1&origin=https%3A%2F%2Fwww.corriere.it%2Fcronache%2F20_marzo_09%2Fcoronavirus-scegliamo-chi-curare-chi-no-come-ogni-guerra-196f7d34-617d-11ea-8f33-90c941af0f23.shtml

[12] Jozsef E Coronavirus en Lombardie : «Dans mon hôpital, il y a environ un ou deux décès par jour». [Coronavirus in Lombardy: “In my hospital there are around one to two deaths per day”]. Libération. 2020;8 French. [Accessed 9 Mar 2020]. Available from: https://www.liberation.fr/planete/2020/03/08/dans-mon-hopital-il-y-a-environ-un-ou-deux-deces-par-jour_1781020

[13] Horowitz J. Italy’s Health Care System Groans Under Coronavirus — a Warning to the World. The New York Times, 12 March 2020. [Accessed 15 March 2020]. Available from: https://www.nytimes.com/2020/03/12/world/europe/12italy-coronavirus-health-care.html

[14] Rhodes A Ferdinande P Flaatten H Guidet B Metnitz PG Moreno RP The variability of critical care bed numbers in Europe. Intensive Care Med. 2012;38(10):1647-53. 10.1007/s00134-012-2627-8 22777516

