# Supplementary material for: Rapidly increasing cumulative incidence of coronavirus disease (COVID-19) in the European Union/European Economic Area and the United Kingdom, 1 January to 15 March 2020
Source: Euro Surveill. 2020 Mar 19;25(11):2000285. doi: 10.2807/1560-7917.ES.2020.25.11.2000285 (PMC7096777; doi:10.2807/1560-7917.ES.2020.25.11.2000285)

This supplementary material is hosted by Eurosurveillance as supporting information alongside the article "Rapidly increasing cumulative incidence of coronavirus disease (COVID-19) in the European Union/ European Economic Area and the United Kingdom, 1 January to 15 March 2020." on behalf of the authors who remain responsible for the accuracy and appropriateness of the content. The same standards for ethics, copyright, attributions and permissions as for the article apply. Eurosurveillance is not responsible for the maintenance of any links or email addresses provided therein.

Supplementary material

Supplementary material 1. Evolution of the 14-day truncated cumulative incidence of COVID-19 cases and incidence of new COVID-19 cases, EU/EEA and the United Kingdom, overall and by country, 25 January*- 15 March 2020.

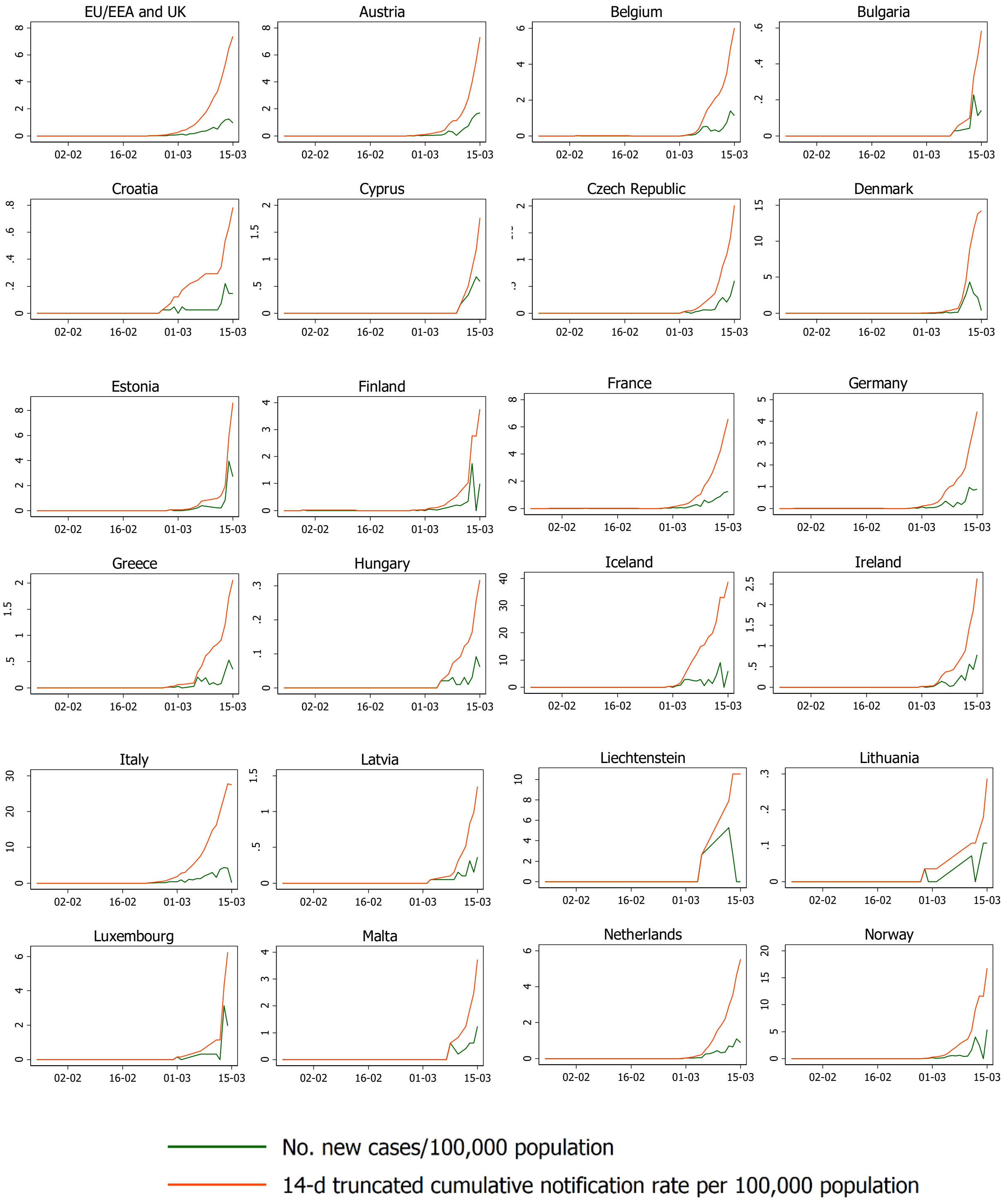

Supplementary material 1 (continued). Evolution of 14-day truncated cumulative incidence of COVID-19 cases and incidence of new COVI-19 cases, EU/EEA and the United Kingdom, overall and by country, 25 January*-15 March 2020.

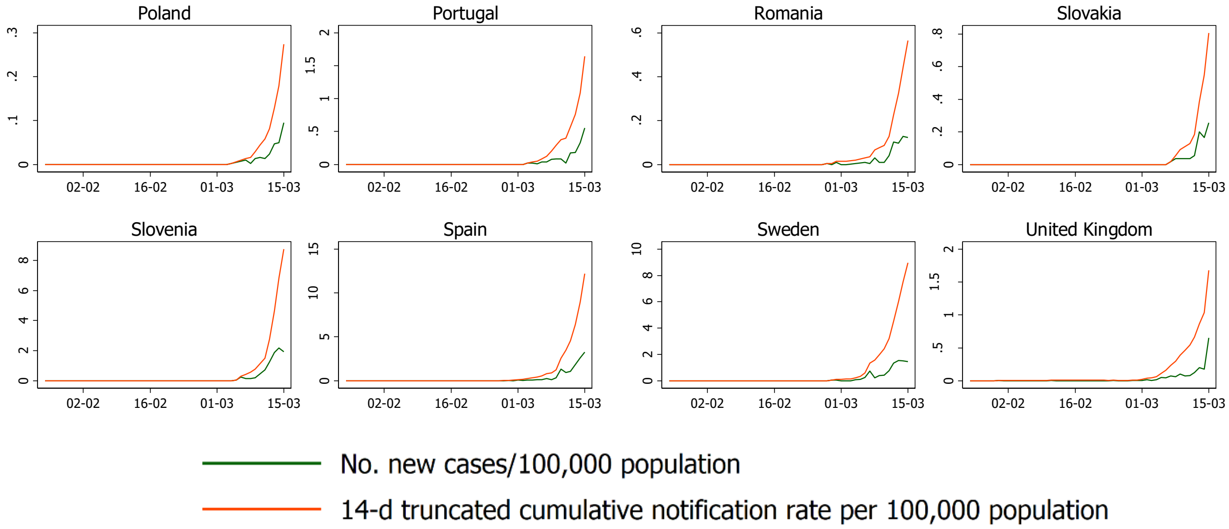

*25 January 2020 is the date when the first three imported cases of COVID-19 were reported in the EU/EEA and UK, by France.

Supplement material 2. 14-day truncated cumulative incidence of COVID-19 cases as at 15 March 2020, by country, EU/EEA and the United Kingdom

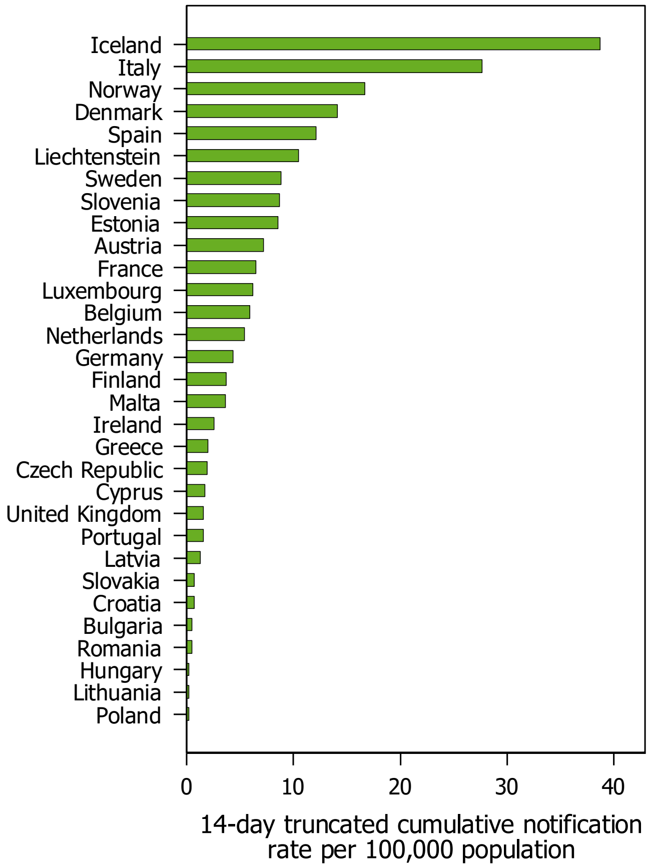

Supplement: Supplementary Material [file 2000285_MONNET_Supplementary_Material.pdf]
